# X-linked genes exhibit skewed expression in Sjogren’s disease (SjD): a further step toward understanding the female predominance of autoimmune disease

**DOI:** 10.1007/s00109-022-02223-1

**Published:** 2022-08-18

**Authors:** Robert Fox

**Affiliations:** Division of Rheumatology, Scripps Memorial Hospital and Research Foundation-Ximed, La Jolla, CA USA

Shaw et al. [1] (https://doi.org/10.1007/s00109-022-02205-3) performed genome-wide, allele-specific profiling from salivary glands derived from mesenchymal cells. The cell lines were derived from patients with Sjogren’s disease (SjD) and were compared to the results from cell lines from females *lacking* autoimmune disease.

In particular, they detected *major differences in the regulation of X-linked genes* from the *female SjD patients*, in comparison to cell lines from *control female subjects*.

- In the cell lines from *control* females, X-linked genes were expressed from paternal and maternal X-chromosomes with the expected maternal: paternal ratio of − 0.5.
- However, in cell lines derived from female patients *diagnosed with SjD*, X-linked genes exhibited preferential expression of one of the two X-chromosomes. The mechanisms underlying this imbalance will be discussed below.

The primary take home lesson is that something interesting is happening to X-chromosome patients with *autoimmunity*, in comparison to *normal* female X-chromosome.

“Lyon Hypothesis” [2, 3] states that *the phenotypic effect of the X chromosome inactivation is the same in the mammalian female as it is in the male which has only one X chromosome*. One out of two X-chromosomes in females is inactivated early in embryonic development. It is called the Barr body.

This study points out a *second* process in which X-chromosomes from females with autoimmune disease exhibit skewed inactivation [4]. This observation provides a mechanism in which the concordance of autoimmune disease in genetically identical twins is only about 20% concordance [5] rather than 100% concordance if only genetic and hormonal factors were needed for clinical disease.

This skewing of X-linked genes may help explain the *marked female predominance of autoimmune disease* by an increase in hormones due to gene dosage effects. Further, some epigenetic process such as an environmental trigger may trigger the start of this pattern of skewing in genetically suspectable individuals.

In early development, the human female chromosome is characterized by monoallelic expression of the non-coding RNA XIST (x-inactive specific transcript) [6]. XIST binds at a specific “x inactivation center,” which in turn initiates the silencing of one X-chromosome. X-chromosome inactivation is microscopically identifiable by the formation of a heterochromatic Barr body carrying the histone H3 modification H3K27me marker, which causes repression of transcription. *Epidemiologic studies have shown that over 80% of autoimmune disease patients are female.* SjD is a chronic autoimmune disease, characterized by aberrant innate and adaptive immune response leading to exocrine gland lymphocytic infiltrates, with an impressive female-to-male ratio of 9:1 [8]. SjD, characteristically, has distinct peaks of disease onset [7]. A *minority* have onset around *pregnancy*. Although SjD can present in children, it is quite unusual before menarche [8]. The *majority of SjD patients* have clinical onset during the *peri-menopausal time period*.

Interestingly, despite the majority of patients experiencing disease onset around the time of menopause, SjD is characterized by autoantibodies (anti-SS A and anti-SS B) that may precede the onset of clinical SjD by many years [8]. Finally, SjD symptoms are affected by pregnancy or with exogenous hormone use. Taken together, these findings indicate sex hormone shifts might contribute to SjD pathogenesis. They are not; however, the whole story X-chromosomes have also been implicated in SjD. In males with SjD, the incidence of co-existent Klinefelter syndrome (XXY) (adversely affecting testicular growth and testosterone production) is markedly increased.

Extensive genome-wide association studies (GWAS) have been published in SjD, and none of the associations comes anywhere close to the female predominance. The usual explanation to medical students is that female preponderance in autoimmune disease is due to Darwinian natural selection that has shaped the female immune system to accept the implantation and gestation of a semi-allogeneic fetus. The hypothesis dates back to the time of Sir Burnet [9], who invoked failure of tolerance at the time of thymic selection process of immune cells in women and was expanded by Sir Medawar [10]. During females’ reproductive and hormonal cycles, some hormonal response genes become “out of balance.” During such events as pregnancy, menopause, or while using birth control, hormones clinically would accelerate the breakdown of tolerance to a specific self-antigen.

Additional evidence has been provided to support this hypothesis by many studies that demonstrated skewed X-linked genes that promote escape from tolerance induction during thymic development, and subsequently play a role in triggering autoimmune disease [11]. Other studies have suggested differences in hormonal and cellular immune responses in women as a result of ovarian steroids estrogen and progesterone [12].

However, this study by Shaw and co-workers makes us ask this question more critically. The potential escape of certain X-linked genes from inactivation in patients with autoimmune disease leads not only to clues for pathogenesis (triggers) but also to *new potential therapeutic targets* [13].

One immediate candidate might be the *Toll-like 7 or Toll-like 8 receptors* which are known to be located on the X-chromosome [14]. However, they did not report skewing of the expression of these Toll-like receptors. However, one intriguing clue was deregulation of the X-linked microRNA miR6891-5p—in autoimmune cells, in comparison to cell lines derived from normal females. The mechanism involves epigenic changes in histone (H3K27me3). Further, they found that unequal silencing is due to a decrease in the XIST level that in turn alters the histone regulation.

This unequal silencing was not an artifact of a single cell line, but was found in 4 differently established salivary mesenchymal cell lines derived from SjD subjects, while *not* observed in 4 *control* female cell lines. Interestingly, the set of silenced genes was not identical in all the SjD cell lines, although overlap was noted (Supplemental Fig. 2 and Supplementary Table). Also, skewing was noted in samples from Systemic Lupus Erythematosus patients (Fig. 5) in comparison to control females.

They have excluded artifacts introduced in maintaining cell lines by allele-specific PCR (Supplementary Fig. 5a).

These intriguing studies have identified a new batch of candidate deregulated genes that have emerged in at least 1 SjD patient (DOCK 11, MECP2, and IRAK1). Other findings such as alteration in H3K27me3 deposition and decreased level of miR6891-5p (a HLA expressed miRNA) may provide further candidates. The role of miR6981 in this inhibition leading to H3K27me3 and the allelic skewing are new challenges for understanding pathogenesis of the female-predominant autoimmunity.

In summary, this article points out possible mechanisms by which environmental triggers can lead to clinically significant autoimmune manifestations in genetically suspectable individuals. In fact, it focuses our attention on the often overlooked observation that these autoimmune diseases occur predominantly in women.
